# Dual-Branch Deep Learning with Dynamic Stage Detection for CT Tube Life Prediction

**DOI:** 10.3390/s25154790

**Published:** 2025-08-04

**Authors:** Zhu Chen, Yuedan Liu, Zhibin Qin, Haojie Li, Siyuan Xie, Litian Fan, Qilin Liu, Jin Huang

**Affiliations:** 1 Department of Medical Engineering, West China Hospital, Sichuan University, Chengdu 610041, China; cz_0220@wchscu.cn (Z.C.); wentuo0516@wchscu.com (H.L.); xiesiyuan@wchscu.com (S.X.); flt@wchscu.com (L.F.); 2 Innovation Institute for Integration of Medicine and Engineering, West China Hospital, Sichuan University, Chengdu 610041, China; liuyuedan0401@wchscu.cn; 3 Chengdu Women’s and Children’s Central Hospital, School of Medicine, University of Electronic Science and Technology of China, Chengdu 611731, China; qinzb1992@hotmail.com

**Keywords:** CT equipment, X-ray tube, remaining useful life, deep learning, residual self-attention, temporal convolutional network

## Abstract

CT scanners are essential tools in modern medical imaging. Sudden failures of their X-ray tubes can lead to equipment downtime, affecting healthcare services and patient diagnosis. However, existing prediction methods based on a single model struggle to adapt to the multi-stage variation characteristics of tube lifespan and have limited modeling capabilities for temporal features. To address these issues, this paper proposes an intelligent prediction architecture for CT tubes’ remaining useful life based on a dual-branch neural network. This architecture consists of two specialized branches: a residual self-attention BiLSTM (RSA-BiLSTM) and a multi-layer dilation temporal convolutional network (D-TCN). The RSA-BiLSTM branch extracts multi-scale features and also enhances the long-term dependency modeling capability for temporal data. The D-TCN branch captures multi-scale temporal features through multi-layer dilated convolutions, effectively handling non-linear changes in the degradation phase. Furthermore, a dynamic phase detector is applied to integrate the prediction results from both branches. In terms of optimization strategy, a dynamically weighted triplet mixed loss function is designed to adjust the weight ratios of different prediction tasks, effectively solving the problems of sample imbalance and uneven prediction accuracy. Experimental results using leave-one-out cross-validation (LOOCV) on six different CT tube datasets show that the proposed method achieved significant advantages over five comparison models, with an average MSE of 2.92, MAE of 0.46, and R^2^ of 0.77. The LOOCV strategy ensures robust evaluation by testing each tube dataset independently while training on the remaining five, providing reliable generalization assessment across different CT equipment. Ablation experiments further confirmed that the collaborative design of multiple components is significant for improving the accuracy of X-ray tubes remaining life prediction.

## 1. Introduction

CT scanners are important medical imaging devices that provide three-dimensional images of internal body tissues through X-ray tomography, which play a key role in disease screening, localization, and diagnosis [1]. However, these critical and expensive medical assets face significant operational challenges, with X-ray tube failures representing the most costly and disruptive maintenance issue. Unexpected tube failures can result in equipment downtime lasting days to weeks, directly impacting patient care delivery and hospital operations. Traditional reactive and preventive maintenance approaches are inadequate for managing such critical equipment, motivating the development of intelligent predictive maintenance solutions specifically designed for medical environments. Currently, healthcare institutions are increasingly focusing on the application of predictive maintenance (PdM) strategies for medical equipment. CT scanners frequently experience downtime due to sudden failures of components such as X-ray tubes, which not only affect the normal healthcare operations of hospitals but also delay timely diagnosis for patients [2]. Traditional preventive maintenance (PvM) strategies have limited ability to handle random sudden failures [3]. Therefore, accurately predicting the remaining useful life of CT tubes helps hospitals develop reasonable tube replacement plans and further reduce the risk of unexpected CT equipment downtime [4].

Predictive maintenance (PdM) has been widely applied in modern industrial production [5], especially in predicting equipment’s remaining useful life (RUL) in engineering, mechanical, and automation applications [6,7]. Equipment maintenance spans the entire lifecycle of the equipment, and a good maintenance strategy can significantly extend equipment lifespan and reduce failure rates [8,9]. PdM, based on historical data collected during equipment operation, utilizes advanced data analysis and machine learning technologies to achieve the precise prediction of equipment RUL, providing a scientific basis for equipment maintenance decisions [10,11].

Compared to traditional physics-based methods, data-driven degradation prediction methods can automatically learn equipment degradation patterns and rules from large-scale heterogeneous data without an in-depth understanding of complex failure mechanisms, thus possessing stronger universality and adaptability [12,13]. With the rapid development of sensor technology, IoT, and big data processing capabilities, the acquisition and storage of large amounts of operational data have become easier, providing a solid foundation for data-driven degradation prediction methods [14,15].

Currently, data-driven degradation prediction methods can be categorized into three main types: statistical learning methods, machine learning methods, and deep learning methods [16,17,18]. Statistical learning methods model the degradation process through probability and statistics theory [19], such as hidden Markov models, Weibull distribution models, Gamma processes [20], Wiener processes [21], and random filtering [22,23]. However, these methods rely on prior assumptions about the degradation process and have limitations in handling complex non-linear relationships, making them difficult to adapt to the multi-stage variation characteristics in CT tube life prediction. Machine learning methods such as support vector machines and random forests extract features from historical data and learn degradation patterns [24]. Wei et al. [25] proposed a method combining support vector regression (SVR) with particle filters to predict the RUL of lithium-ion batteries. This method could improve prediction accuracy to some extent but still struggles to capture complex temporal dependencies when processing long sequence data, making it difficult to learn features directly from raw data. Deep learning methods such as long short-term memory (LSTM) networks and convolutional neural networks can automatically learn complex temporal features from raw data, achieving end-to-end degradation prediction [26]. For example, Ciani et al. [27] used LSTM and a single exponential degradation model to accurately estimate the RUL of lithium batteries in wireless sensor networks based on a limited number of data records. Ma et al. [28] combined the CNN with feature extraction capability and LSTM with recurrent memory capability to carry out bearing degradation trend prediction. Deep learning methods, such as LSTM networks and convolutional neural networks, can automatically learn complex temporal features from raw data, achieving end-to-end degradation prediction. However, single deep learning models have limitations in CT tube life prediction. For example, LSTM may encounter gradient vanishing or explosion problems with long sequences, while CNN lacks the ability to model temporal dependencies when processing time series data.

Despite significant progress in data-driven degradation prediction methods, the development of a prediction method suitable for X-ray tubes of CT equipment still faces enormous challenges [29]. On the one hand, there are evident issues of data non-linearity and multi-phase characteristics for the collected CT tube data. The tube lifespan data exhibits complex non-linear degradation patterns and shows different degradation features at different usage stages. There are significant differences in data distribution between early stages, mid-use periods, and near-failure phases, making it difficult for a traditional model to capture the variation patterns throughout the entire lifecycle. Additionally, factors such as the uncertainty of the various working environments, parameter fluctuations, and load changes further increase the complexity of data modeling, leading to insufficient prediction accuracy of traditional or single-stage models in practical applications. On the other hand, from the perspective of prediction model construction, traditional static model structures cannot dynamically adjust prediction strategies based on different data characteristics, lacking adaptive mechanisms to handle different types of temporal patterns [30]. The architecture of a single model is also unable to process different types of temporal patterns simultaneously. For example, a pure LSTM model encounters gradient vanishing or exploding problems with long sequences, making it difficult to capture long-term dependencies. A pure temporal convolutional network (TCN) model, although capable of handling long sequences, lacks the ability to process sequential and state information. In addition, simple loss functions result in the model being insensitive to state transitions at turning points in the data, and the use of standard MSE or MAE loss functions fails to distinguish the importance of different types of samples. Fixed-weight loss functions also cannot adapt to the requirements of different training stages [31,32].

To address the above issues, this paper proposes a CT tube life intelligent prediction architecture based on a dual-branch neural network (DDLNet). Firstly, an adaptive phase-aware framework is applied to identify the different phases of tube, enabling the model to adaptively adjust prediction strategies according to the current phase, which can address the problem of insufficient model adaptability. Secondly, a dual-branch collaborative architecture is applied to enhance the modeling capability of long-term dependencies from temporal data, effectively capturing non-linear changes in the degradation phase and overcoming the limitations of a single model. Finally, a dynamically weighted triplet mixed loss function is designed to adaptively adjust the weight ratios of different prediction tasks, solving the problems of sample imbalance and uneven prediction accuracy.

Moreover, the evaluation methodology presents another critical challenge in CT tube RUL prediction research. Due to the limited availability of complete lifecycle data from medical equipment and the substantial costs associated with CT tube replacement, most studies rely on small datasets. This necessitates robust evaluation strategies that can provide reliable performance assessment despite limited sample sizes. Traditional train–test splits may lead to optimistic performance estimates when dealing with such constrained datasets, making it essential to employ rigorous cross-validation methodologies that ensure model generalization across different equipment and operating conditions.

Despite these advances, several critical research gaps remain in CT tube RUL prediction. First, existing single-model approaches cannot effectively capture the multi-phase degradation characteristics of CT tubes, as they lack adaptive mechanisms to handle the distinct patterns between stable and degradation phases. Second, current methods fail to synergistically combine long-term dependency modeling with multi-scale temporal feature extraction, limiting their effectiveness in complex industrial environments. Third, traditional loss functions do not adequately address the sample imbalance issues inherent in CT tube datasets, where stable phase data significantly outnumber degradation phase samples.

To better clarify our contributions, the key innovations of this work include the following.

(1) A dual-branch neural network architecture that integrates RSA-BiLSTM with D-TCN for CT tube RUL prediction. The RSA-BiLSTM branch uses a CNN-QKV generator to transform input sequences into query, key, and value representations and employs a forward-looking attention mechanism where query vectors at time step *t* + 1 interact with key vectors at time step *t* to capture state transitions. The D-TCN branch uses dilated convolutions with dilation rates of 1, 2, 4, and 8 to extract multi-scale temporal features. This dual-branch design addresses gradient vanishing problems in single LSTM models and temporal dependency limitations of pure CNN models.

(2) An adaptive phase-aware framework with dynamic stage detection for binary CT tube degradation states. The framework identifies the stable phase (≥30 days) and the degradation phase (<30 days) to enable adaptive prediction strategies across different operational states.

(3) A dynamically weighted triplet mixed loss function combining base value loss, phase classification loss, and smoothness loss. The dynamic weighting mechanism adjusts each component’s contribution based on real-time metrics, including prediction error, change point density, and prediction smoothness. This automatically adapts the loss function emphasis during training to handle sample imbalance and improve prediction accuracy across different RUL ranges.

The remainder of this paper is organized as follows: Section 2 introduces the proposed method and related knowledge, Section 3 introduces the experimental dataset sources and data processing. Section 4 demonstrates the superiority and effectiveness of the proposed method through comparison with existing methods and ablation experiments. Section 5 concludes the paper.

## 2. Experiment Dataset and Preprocessing Step

### 2.1. Data Description

The Internet of Medical Things (IoMT) system achieves the collection, processing, and analysis of medical data through a multi-layer architecture, including sensor layer, transmission layer, and application layer [33,34]. The sensor layer consists of various sensors monitoring patient physiological data and medical device status. The transmission layer utilizes wireless communication protocols to transmit data to central servers. The application layer is responsible for data storage, analysis, and ensuring secure access.

All data used in this research comes from the IoMT system of West China Hospital. The research team selected 6 X-ray tubes with complete lifecycles from 6 CT devices for RUL prediction research. Table 1 shows the specific information of 6 X-ray tubes, including CT number, tube number, start time, end time, and data volume.

The collected data includes a total of 45 parameters, categorized into device identification, time information, device control parameters, interruption information, and lifespan-related parameters. These parameters reflect various aspects of CT tube operation and degradation, including scan counts that indicate cumulative usage, voltage fluctuations that affect tube stress, temperature measurements that reflect thermal conditions, and arc events that signal electrical anomalies. Data sampling frequency is not fixed, with sampling intervals ranging from 20 to 60 s. Among these, the device control parameters include 5 variables: “focus”, “kind”, “mode”, “region”, and “dom_type”.

The experiment primarily uses the control parameters of 6 tubes. “Focus” refers to the position where the electron beam hits the target material, with the values “3” and “1” representing large focus and small focus, respectively. The data shows that small focus is used more frequently, indicating a greater demand for high-resolution imaging in clinical applications. Scan types (“kind”) mainly include the SEQ, SPI, TOP, and ROT modes. Exposure modes (“mode”) primarily include “A”, “AB”, and “B”, with this research mainly using the “A” mode. Scanning regions (“region”) are divided into “head” and “body”, with the data showing that body scans are predominant, which aligns with clinical practice as body scans encompass chest, abdomen, and pelvic examinations. “Dom_type” represents the tube current dynamic adjustment mode, including “AEC” (360° rotation tube current mode), “ZEC” (Z-direction automatic tube current adjustment mode), and “No” (standard tube current mode without adjustment). These detailed parameter data provide a solid foundation for studying the performance degradation characteristics of CT X-ray tubes and predicting their remaining useful life.

The CT tube lifespan data in the dataset is divided into two phases: the stable phase and the degradation phase. The stable phase is characterized by a lifespan value of 30 days or more, indicating that the equipment is operating normally. The degradation phase is marked by a lifespan value of less than 30 days, indicating that the performance of the equipment is declining and approaching failure. This binary phase division reflects the characteristic changes of CT tubes at different usage stages and provides a basis for the model to distinguish between different working states, thereby enhancing prediction accuracy and adaptability.

### 2.2. Preprocessing Step

The initial phase requires collecting CT operation log data through the medical IoMT system. Our dataset contains complete lifecycle data for 6 CT X-ray tubes, with each tube generating approximately 45 parameters. These parameters can be divided into three main categories: 8 parameters related to device identification and time information, 7 parameters related to device control and operational interruptions, and 30 parameters directly related to tube lifespan characteristics.

For effective analysis, we first apply min–max normalization to 30 lifespan-related parameters to address the issue of varying measurement units and scales between different parameters, which can not only improve the prediction performance of the model but also accelerate convergence during the training process. The *min–max* normalization is calculated as follows:

(1) $$x_{norm}=\frac{x-x_{min}}{x_{max}-x_{min}}$$

After normalization, feature engineering is performed to derive new features from the normalized parameters. This process includes creating interaction terms between existing features through basic arithmetic operations (such as differences, sums, products, and ratios). These derived features capture complex relationships that might not be evident in the original parameters, effectively revealing hidden patterns in the data and enhancing the ability of the model to recognize non-linear relationships.

To determine the most relevant parameters for predicting RUL, this research employs Pearson correlation analysis. This technique evaluates the linear relationship between each feature and the target variable. The Pearson correlation coefficient ranges from −1 to 1, with values closer to either extreme indicating stronger relationships. The Pearson correlation coefficient is defined as

(2) $$r_{xy}=\frac{\Sigma_{i=1}^{n}(x_{i}-\bar{x})(y_{i}-\bar{y})}{\sqrt{\Sigma_{i=1}^{n}{\left(x_{i}-\bar{x}\right)}^{2}}\sqrt{\Sigma_{i=1}^{n}{\left(y_{i}-\bar{y}\right)}^{2}}}$$

This research sets a correlation threshold of 0.75, selecting only those features with absolute correlation coefficients exceeding this value. The Pearson correlation method provides computational efficiency and direct interpretability, allowing the research to quickly identify the most important predictors without involving the complexity or potential instability of other techniques. The correlation analysis reveals several highly correlated features, including parameters related to scan counts, voltage fluctuations, temperature measurements, and arc events.

Finally, we apply sliding window processing to the screened features and data, but original window data often contains significant fluctuations that may mask potential degradation trends. To address this issue, two key processing techniques are applied.

Downsampling: By selecting points at regular intervals (sampling interval T = 20), data density is reduced, which helps smooth short-term fluctuations.

Exponential Moving Average (EMA): Applying EMA smoothing operations further reduces noise and highlights gradual degradation patterns.

## 3. The Proposed Method

### 3.1. Method Overview

**Figure 1:**
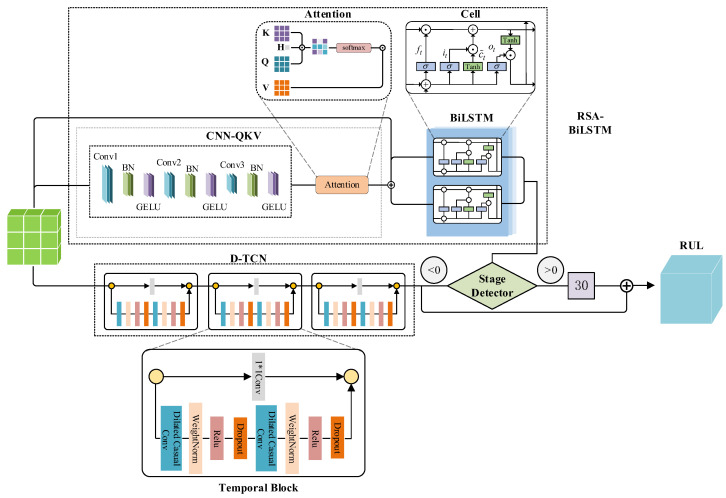
Structure of DDLNet.

This paper develops a DDLNet model to address the problem of predicting the RUL of CT X-ray tubes with multiple working conditions. This architecture integrates the strengths of RSA-BiLSTM and D-TCN and further introduces a dynamic phase detector and dynamically weighted triplet mixed loss function to achieve high-precision prediction. The core of this architecture lies in identifying the binary nature of tube lifespan states: the stable phase (lifespan value of 30 days or more) and the degradation phase (lifespan value less than 30 days). The structure of DDLNet is shown in Figure 1.

Firstly, the processed feature sequences are simultaneously fed into the RSA-BiLSTM and D-TCN branches for parallel feature extraction. The former captures long-term temporal dependencies from stable-phase features through a CNN-QKV generator and BiLSTM, while the latter utilizes multi-layer dilated convolutions to build multi-scale receptive fields and address non-linear changes in degradation phase. Secondly, based on the feature representation from the RSA-BiLSTM branch, the dynamic phase detector generates a state confidence score indicating the current working phase of the tube. Finally, the system adaptively integrates the prediction results from both branches based on the output. When the detection value is greater than 0, it is determined to be in the stable phase and directly outputs 30. Otherwise, it adopts the prediction value from the D-TCN branch as the final output. During the training process, this paper designs a dynamically weighted triplet mixed loss function including base value loss, phase loss, and smoothness loss. The model optimizes prediction accuracy, phase classification accuracy, and prediction curve smoothness simultaneously through the dynamically weighted triplet mixed loss function, combined with a phased pre-training strategy to improve the convergence efficiency and prediction performance of the model. To ensure robust model evaluation and validate generalization capability across different CT equipment, this research employs a LOOCV strategy throughout the experimental validation process.

### 3.2. RSA-LSTM Branch

In the hybrid model, the RSA-BiLSTM branch is specifically used to capture long-term dependencies and key state transition points in CT tube time series data. RSA-LSTM combines the advantages of convolutional neural networks, self-attention mechanisms, and bidirectional LSTM, forming a new architecture capable of precisely identifying specific patterns.

In the RSA-BiLSTM branch, the first key component is the CNN-QKV generator, which extracts features from the input sequence and generates query (Q), key (K), and value (V) representations through convolutional neural networks. This component is designed as follows.

Given an input sequence $X_{t}\in {\mathbb{R}}^{B\times L\times D}$, where *B* is the batch size, *L* is the sequence length, and *D* is the feature dimension, the CNN-QKV generator first performs dimension conversion on the input:

(3) $$X_{trans}=X_{t}.transpose(0,2,1)\in {\mathbb{R}}^{B\times D\times L}$$

Then features are extracted through a three-layer one-dimensional convolution Conv1D, as shown in Equations (2)–(4).

(4) $$F_{1}=\sigma \left(BN\left(Conv1D(X_{trans})\right)\right)$$

(5) $$F_{2}=\sigma \left(BN\left(Conv1D(F_{1})\right)\right)$$

(6) $$F=\sigma \left(BN\left(Conv1D(F_{2})\right)\right)\in {\mathbb{R}}^{B\times H\times L}$$

where σ is the Gaussian error linear unit (GELU) activation function. *BN* is batch normalization. *H* is the hidden-layer dimension. Finally, QKV representations are generated through three independent 1 × 1 convolution projections.

(7) $$Q=W_{q}{\left(F\right)}^{T}\in {\mathbb{R}}^{B\times L\times H}$$

(8) $$K=W_{k}{\left(F\right)}^{T}\in {\mathbb{R}}^{B\times L\times H}$$

(9) $$V=W_{v}{\left(F\right)}^{T}\in {\mathbb{R}}^{B\times L\times H}$$

Next, we designed a special recursive self-attention mechanism that enhances the perception of temporal patterns by the model through information interaction across the time dimension, which is beneficial to adapt to the characteristics of temporal prediction tasks. The self-attention module processes *Q*, *K*, and *V* by time step. For each time step *t*, we employ a “forward-looking” mechanism, where the query vector of time step *t* + 1 interacts with the key vector of the current time step *t*.

(10) $$a_{t}=\frac{Q_{t+1}K_{t}^{T}}{\sqrt{H}}$$

(11) $$\alpha_{t}=softmax(a_{t})$$

(12) $$c_{t}=\alpha_{t}V_{t}$$

where $\sqrt{H}$ is a scaling factor used to prevent gradient vanishing problems and $\alpha_{t}$ is the attention weight. $c_{t}$ is the context vector obtained through the attention mechanism. This forward-looking attention mechanism enables the model to better capture state transition information in the sequence.

After obtaining the attention context vector, we perform feature fusion of the original input features $X_{t}$ with the context vector c.

(13) $$f_{t}=LayerNorm\left(W_{f}[X_{t};c_{t}]+b_{f}\right)$$

(14) $$X_{t}^{\prime }=X_{t}+\lambda \cdot f_{t}$$

where $W_{f}$ and $b_{f}$ are learnable parameters. λ is the attention scaling factor (learned through parameters). $\left[X_{t},c_{t}\right]$ represents vector concatenation. Through residual connections, we enhance the feature expression capability of the model while preserving the original information.

After feature enhancement, $X_{t}^{\prime }$ is input into a bidirectional *LSTM* network.

(15) $${\vec{h}}_{t},{\vec{c}}_{t}=LSTM_{forward}\left(X_{t}^{\prime },{\vec{h}}_{t-1},{\vec{c}}_{t-1}\right)$$

(16) $${\overset{\leftarrow }{h}}_{t},{\overset{\leftarrow }{c}}_{t}=LSTM_{forward}\left(X_{t}^{\prime },{\overset{\leftarrow }{h}}_{t-1},{\overset{\leftarrow }{c}}_{t-1}\right)$$

where the forward *LSTM* captures historical information from the start position to the end position of the sequence. The backward *LSTM* processes from the sequence end position to the start position, capturing future information. ${\vec{h}}_{t}$ and ${\overset{\leftarrow }{h}}_{t}$ represent the hidden states of the forward and backward *LSTM* at time step t, respectively. ${\vec{c}}_{t}$ and ${\overset{\leftarrow }{c}}_{t}$ represent the corresponding cell states.

After obtaining the hidden states of the bidirectional LSTM, we merge the forward and backward hidden states through linear mapping:

(17) $$h_{t}=W_{o}[{\vec{h}}_{t};{\overset{\leftarrow }{h}}_{t}]+b_{o}$$

Here, $\left[{\vec{h}}_{t};{\overset{\leftarrow }{h}}_{t}\right]$ represents vector concatenation. $W_{o}$ and $b_{o}$ are learnable parameters. By combining information from both directions, the model can utilize both past and future contexts to obtain a more comprehensive sequence representation.

At the same time, to improve the robustness and generalization capability of the model, we employ a two-layer stacked bidirectional LSTM structure to enhance representation capability, and further apply dropout between LSTM layers to prevent overfitting [35,36].

(18) $$h_{t}^{\left(1\right)}=BiLSTM^{\left(1\right)}(X_{t}^{\prime })$$

(19) $${\tilde{h}}_{t}^{\left(1\right)}=Dropout\left(h_{t}^{\left(1\right)},p\right)$$

(20) $$h_{t}^{\left(2\right)}=BiLSTM^{\left(2\right)}({\tilde{h}}_{t}^{\left(1\right)})$$

### 3.3. TCN Branch

The other branch is TCN structure, which adopts a dilated convolutional architecture that effectively expands the receptive field and preserves local features, capable of capturing multi-scale temporal dependencies. Given an input sequence $X_{t}\in {\mathbb{R}}^{B\times L\times D}$, where *B* is the batch size, *L* is the sequence length, and *D* is the feature dimension. The *TCN* branch first performs dimension conversion.

(21) $$X_{trans}=X_{t}.transpose(0,2,1)\in {\mathbb{R}}^{B\times D\times L}$$

This conversion facilitates subsequent convolutional operations. The *TCN* branch consists of multiple stacked temporal blocks, with the overall structure being in Equation (20).

(22) $$Y=TCN(X)=W_{o}\cdot TCN_{net}{\left(X_{trans}\right)}^{T}+b_{o}$$

where $W_{o}$ and $b_{o}$ are output layer parameters. $TCN_{net}$ represents the main body of the TCN network. To enhance the representation capability and training stability of TCN, this research improves the traditional temporal convolutional block by introducing more efficient residual connection structures and regularization techniques. Each temporal convolutional block contains of two layers of dilated convolution, structured as follows:

(23) $$Z_{1}=Conv1D(X_{i},k,d_{i},p_{i})$$

(24) $$Z_{2}=BatchNorm(Z_{1})$$

(25) $$Z_{3}=ReLU(Z_{2})$$

(26) $$Z_{4}=Dropout(Z_{3},p=0.2)$$

(27) $$Z_{5}=Conv1D(Z_{4},k,d_{i},p_{i})$$

(28) $$Z_{6}=BatchNorm(Z_{5})$$

(29) $$Z_{7}=Dropout(Z_{6},p=0.2)$$

(30) $$X_{i+1}=ReLU\left(Z_{7}+Residual(X_{i})\right)$$

Here, the residual connection is a key design element that allows information to flow directly from input to output, solving the gradient vanishing problem in deep network training. The basic idea of residual connection is to create a “shortcut” that adds the input directly to the output of the convolutional layer. This residual function is defined by Equations (29) and (30), respectively.

If the number of channels in $X_{i}$ and $Z_{7}$ are the same,

(31) $$Residual(X_{i})=X_{i}$$

If the number of channels in $X_{i}$ and $Z_{7}$ are different,

(32) $$Residual(X_{i})=Conv1D(X_{i},kernel_{size}=1,stride=1)$$

Here, 1 × 1 convolution is used to adjust the number of channels without changing the sequence length. This residual connection allows the network to learn new features or preserve original features. As network depth increases, if adding more layers no longer improves performance, residual connection allows additional layers to approximate identity mappings.

To enable the model to simultaneously attend to local details and global trends, we introduce a multi-scale feature fusion mechanism to establish skip connections between different dilation layers, which can integrate features from different receptive field levels.

(33) $$F_{multi}=\sigma \left(\sum_{i=0}^{2}W_{i}\cdot F_{i}\right)$$

where $F_{i}$ represents the output features of the i-th dilated convolutional layer. $W_{i}$ is the corresponding weight parameter. σ is the activation function. These features are then transformed into prediction values through a specially designed output mapping layer.

(34) $$Y=W_{o}\cdot F_{multi}[:,-1,:]+b_{o}$$

### 3.4. Model Loss Function

To address the issue of prediction accuracy differences across different ranges in CT tube RUL prediction, this paper proposes a mixed loss function:

(35) $$L_{total}=\alpha \cdot L_{value}+\beta \cdot L_{stage}+\gamma \cdot L_{smooth}$$

where α,β and γ are dynamically adjusted weight coefficients that control the contribution of each component loss. Unlike traditional fixed-weight methods, we introduced an adaptive dynamic weight adjustment mechanism based on data characteristics:

(36) $$\alpha =\alpha_{base}\cdot (1+sigmoid(diff_{mean}-1))$$

(37) $$\beta =\beta_{base}\cdot (1+sigmoid(density_{change}\cdot 10-5))$$

(38) $$\gamma =\gamma_{base}\cdot (1+sigmoid(smoothness-0.5))$$

where $diff_{mean}$ is the average difference between predicted and actual values. When prediction error increases, $diff_{mean}$ will rise. And through the sigmoid function, α will also increase, thereby enhancing the weight of basic prediction loss. $density_{change}$ is the change point density (defined as the ratio of the number of change points to the total number of samples). We multiply it by 10 and subtract 5 to map typical distributions of $density_{change}$ values to the sensitive region of the sigmoid function, making the adjustment more effective. smoothness is the average value of differences between consecutive prediction samples. When the prediction results fluctuate significantly, smoothness increases, and γ correspondingly increases, causing the model to focus on the smoothness of the prediction curve.

The base value loss uses weighted mean squared error, assigning different importance to different target values:

(39) $$L_{value}=\frac{1}{n}\sum_{i=1}^{n}w_{i}{\left(pred_{i}-target_{i}\right)}^{2}$$

The phase loss is designed for the classification task, aimed at optimizing the ability of the model to recognize the characteristic value of 30:

(40) $$L_{stage}=\frac{1}{n}\sum_{i=1}^{n}{\left(stage_{prob}^{i}-true_{stage}^{i}\right)}^{2}$$

The smoothness loss is specifically designed for the stability in the 30 value region, ensuring smooth changes in prediction results between adjacent time steps:

(41) $$L_{smooth}=\frac{1}{m}\sum_{i=1}^{n}{\left(pred_{i+1}-pred_{i}\right)}^{2}\cdot I$$

### 3.5. Phase Detector Design and Phased Training Strategy

Since the labeled data has been divided, it exhibits a clear binary phase characteristic: a stable period (value equal to 30.0) and a degradation period (value less than 30.0). To distinguish between these two phases and improve prediction accuracy, we design a dedicated phase detector.

(42) $$Stage(x)=\tan h\left(W_{2}\cdot LeakyReLU(W_{1}\cdot x+b_{1})+b_{2}\right)$$

where $x\in {\mathbb{R}}^{D}$ is the feature vector output from the RSA-LSTM branch and $W_{1}$ and $W_{2}$ are weight matrices. $b_{1}$ and $b_{2}$ are bias terms. When $Stage\left(x\right)>0$, it is determined to be in the stable period, whose prediction value is 30.0, When $Stage\left(x\right)\leq 0$, it is determined to be in the degradation period, and the prediction value adopts the prediction result from TCN branch. The choice of tanh as the activation function considers its zero-centered nature. The introduction of LeakyReLU is to avoid the gradient vanishing problem in the negative value region.

Next, this paper adopts a phased pre-training strategy. For the RSA-LSTM branch, we use stable period data samples for targeted pre-training, while the TCN branch uses degradation period data samples for targeted pre-training. Based on the standard MSE loss function, both branches’ pre-training processes use the AdamW optimizer and OneCycleLR learning rate scheduler with a cosine annealing strategy.

## 4. Case Study

### 4.1. Evaluation Metrics

To evaluate the performance of the RUL model, this research selects three key evaluation metrics, i.e., Mean Squared Error (MSE), Mean Absolute Error (MAE), and Coefficient of Determination (R^2^). These metrics reflect the prediction accuracy of the model from different perspectives, as follows:

1. Mean Squared Error (MSE): MSE is particularly suitable for RUL prediction as it heavily penalizes large prediction errors, which are critical in maintenance planning. In CT tube maintenance, a significant prediction error (e.g., predicting 20 days when actual RUL is 5 days) could lead to equipment failure and patient care disruption. *MSE* is calculated as Equation (45):
  (43) $$MSE=\frac{1}{n}\sum_{i=1}^{n}{\left(y_{i}-{\hat{y}}_{i}\right)}^{2}$$
  where $y_{i}$ is the actual value, ${\hat{y}}_{i}$ is the predicted value, and n is the sample size. *MSE* evaluates the performance of prediction models through squared errors, which is sensitive to large errors.
2. Mean Absolute Error (MAE): MAE provides an intuitive understanding of average prediction accuracy in the same units as the target variable (days). This is directly interpretable by maintenance personnel who need to understand the typical prediction uncertainty when planning maintenance schedules. Unlike MSE, MAE is less sensitive to outliers, providing a robust measure of typical model performance. MAE calculated as
  (44) $$MAE=\frac{1}{n}\sum_{i=1}^{n}|y_{i}-{\hat{y}}_{i}|$$
3. Coefficient of Determination (R^2^): R^2^ indicates how well the model explains the variance in RUL values, which is crucial for assessing model reliability across different CT tubes and operating conditions. In medical equipment maintenance, understanding the proportion of RUL variance that can be explained by the model helps in risk assessment and decision-making confidence. *R*^2^ is calculated as
  (45) $$R^{2}=1-\frac{\Sigma_{i=1}^{n}{\left(y_{i}-{\hat{y}}_{i}\right)}^{2}}{\Sigma_{i=1}^{n}{\left(y_{i}-\bar{y}\right)}^{2}}$$
  where $\bar{y}$ is the mean of the actual values. The *R*^2^ value typically ranges between 0 and 1, with values closer to 1 indicating better model fit, meaning the model can explain more variance in the dependent variable. These three metrics provide complementary perspectives: MSE emphasizes accuracy in critical situations, MAE provides practical interpretability, and R^2^ assesses overall model reliability.

To ensure robust evaluation and avoid overfitting, this study employs a LOOCV strategy. Given the limited number of CT tubes (six tubes), LOOCV provides the most comprehensive evaluation by using each tube as a test set while training on the remaining five tubes. This approach ensures that the model’s performance is evaluated on completely unseen data from different CT scanners, providing a more realistic assessment of the model’s generalization capability across different equipment and operating conditions. While LOOCV provides rigorous evaluation within our dataset, it cannot address the fundamental limitation of domain diversity. The claims of generalizability across different healthcare institutions and CT equipment types remain insufficiently substantiated.

### 4.2. Comparative Experiments

The experiments are conducted on a system with the following configuration: a Windows 10 (22H2) operating system, equipped with an Intel^(R)^ i7-9750H processor and an Nvidia RTX2060 graphics card. The software environment included Python 3.12.1 and PyTorch 2.3.1 for implementing and training the neural network models. All experimental results reported in this study are obtained using LOOCV. For each iteration, one tube dataset is held out as the test set, while the remaining five tube datasets are used for training and validation. This process is repeated six times, ensuring each tube serves as the test set exactly once. The reported metrics (MSE, MAE, and R^2^) represent the average performance across all six cross-validation folds. To evaluate the performance of DDLNet in the CT tube RUL task, this research compared it with five network models, i.e., LSTM, Transformer, CNN-BiLSTM-Attention, CNN-LSTM, and CNN-LSTM-Attention.

Table 2 shows the MSE, MAE, and R^2^ metric values of each model on six different CT tube datasets (Tube 1–6). From the experimental data, DDLNet obtains the best performance on the MSE, MAE, and R^2^ metrics. Notably, on Tube 5, other models generally have low R^2^ values (such as LSTM at 0.0018, CNN-BiLSTM-Attention at 0.0643), while DDLNet reaches a 0.8576 R^2^ value. On all datasets, the R^2^ of DDLNet remained above 0.62, while other models had multiple instances below 0.25. The inclusion of standard deviations reveals that DDLNet demonstrates not only superior mean performance but also the lowest variability across different CT tubes (std: 1.39 for MSE, 0.18 for MAE, 0.10 for R^2^), indicating more consistent and reliable predictions compared to baseline methods. Through comparative analysis, DDLNet showed good performance on all three evaluation metrics, especially when handling complex datasets such as Tube 4 and Tube 5, maintaining relatively stable prediction performance with lower MSE and MAE and higher R^2^.

**Figure 2:**
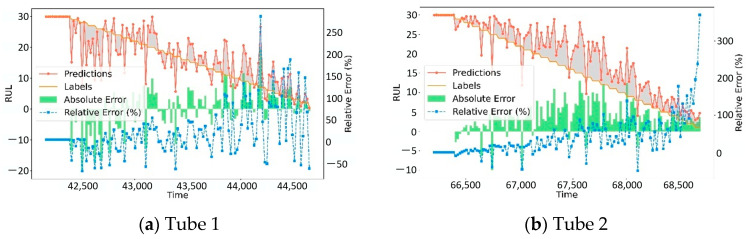

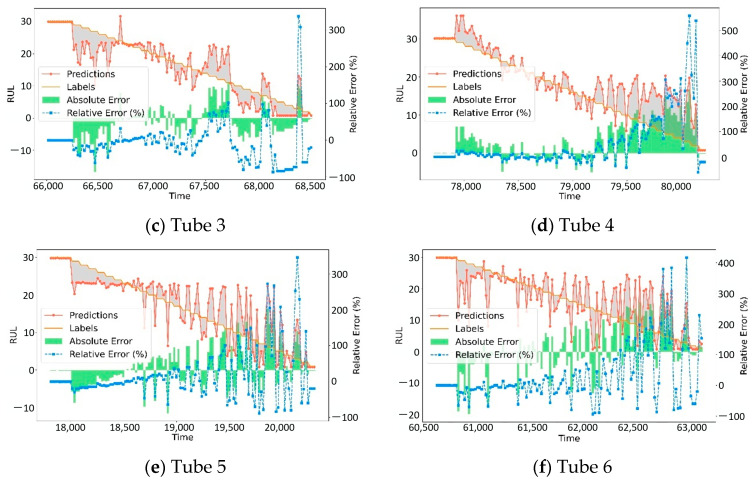
Prediction results.

To visually demonstrate the performance of the DDLNet model in the CT tube RUL prediction, Figure 2 shows the prediction results, actual label values, absolute errors, and relative errors on six test datasets. The red line represents the predicted values, the orange line represents the actual label values, the green bars represent absolute errors, and the blue dashed line represents relative errors. From the visualization results, it can be observed that the prediction values can generally track the changes in the actual RUL values. At most time points, the differences between predicted and actual values are small, with absolute errors generally kept at low levels. The model shows high prediction accuracy in the mid-life stage of CT tubes (RUL values between 10–25). It is worth noting that in all six datasets, when the RUL value is low (i.e., when the tube is approaching the end of its life), the relative error increases. This is mainly because the RUL value itself is small, so even small absolute errors can lead to large relative error percentages. When the RUL approaches 5 or below, increased fluctuations in relative error can be observed, occasionally exceeding 200% relative error peaks. Additionally, in the initial phase of the time series (RUL around 30), the fit between the predicted values of the model and actual values is generally good, with consistently small absolute errors. As time progresses, the prediction curve can capture the downward trend of actual RUL values. Overall, the DDLNet model demonstrates good prediction capability across all six test datasets, effectively tracking the changing trends in CT tubes’ remaining life.

**Figure 3:**
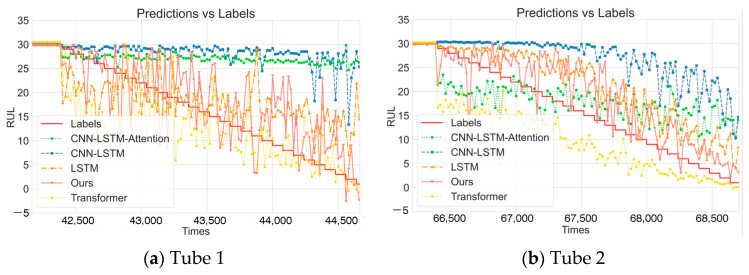

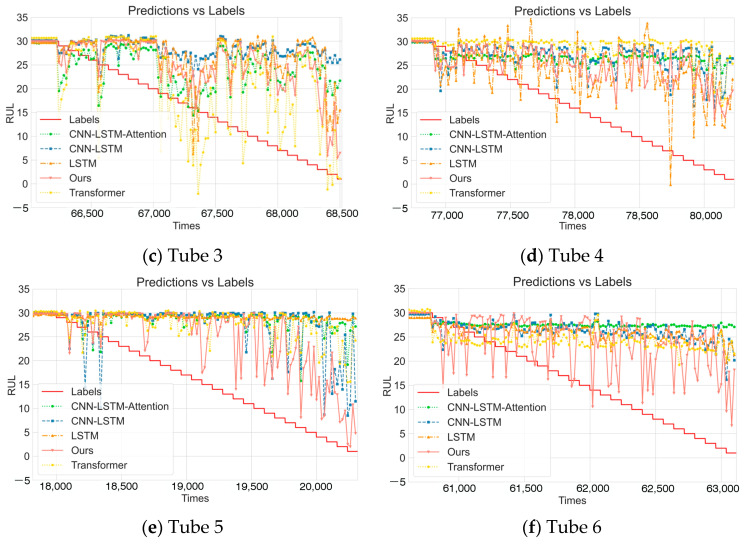
Comparison of different models.

To further visually compare the differences in prediction capabilities between DDLNet and comparison methods, Figure 3 shows the comparison of prediction results and actual label values for various models on six test datasets. Overall, the DDLNet model achieved significant advantages in MSE, MAE, and R^2^ evaluation metrics, particularly showing higher prediction accuracy and stability when handling complex datasets. Tube 4 presents a special case where all comparative models show obvious prediction stability issues in the high RUL region (20–30), with predicted values exhibiting large fluctuations around the actual values. While the DDLNet model also shows some fluctuations, it can better reflect the downward trend of RUL; especially in the region where RUL is below 15, its prediction accuracy is significantly better than those of other models. This indicates that the DDLNet model can effectively address the challenges posed by the multi-phase variation characteristics of CT tubes, providing reliable CT tube remaining life predictions for medical institutions and helping to formulate scientific maintenance plans.

### 4.3. Ablation Study

**Figure 4:**
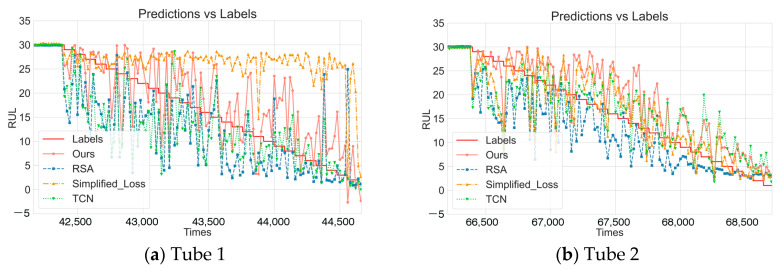

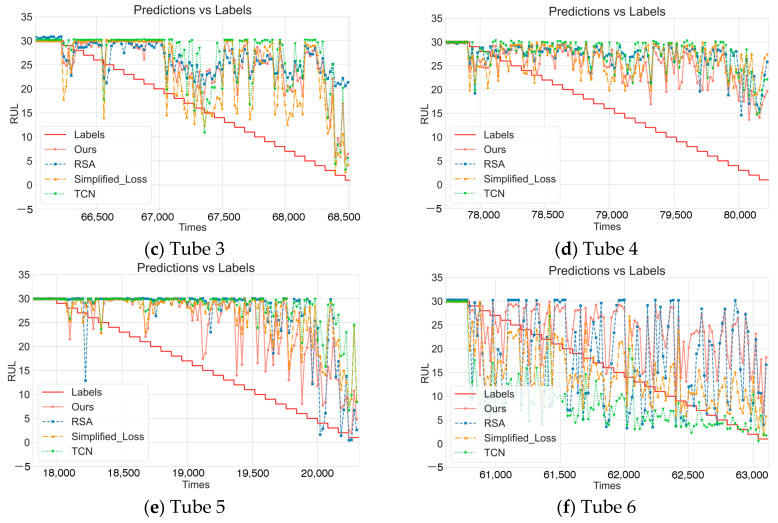
Ablation experiments.

To further verify the contribution of each component of DDLNet, this research designed a series of ablation experiments using the same LOOCV strategy. Figure 4 shows the comparison of prediction performance between the complete DDLNet model (marked as “Ours”) and variant models with specific components removed or modified on six test datasets. This experiment mainly examined the following variants:

1. No pre-training model (No_Pretraining): Removed the pre-training phase, directly training with randomly initialized model parameters to verify the impact of the pre-training strategy on model performance.
2. Residual self-attention model (RSA): Only retained the RSA-BiLSTM branch of DDLNet to evaluate the effectiveness of the RSA-BiLSTM branch acting alone.
3. Simplified loss function model (Simplified_Loss): Used standard mean squared error (MSE) to replace the composite loss function proposed in this paper, removing the relative error term to verify the impact of loss function design on prediction accuracy.
4. Temporal convolutional network model (TCN): Only retained the TCN branch to evaluate the effectiveness of the TCN branch acting alone.

According to the data in Table 3, there are significant performance differences between the variant models and the complete DDLNet. Regarding the pre-training strategy, the No_Pretraining variant has an average MSE of 4.5034, higher than DDLNet’s 2.8016, and its average R^2^ is 0.6479, lower than DDLNet’s 0.7714. Specifically for each dataset, on Tube 3, the MSE of the No_Pretraining model is 3.8784, 71.9% higher than DDLNet (2.2568), and on Tube 4, the MSE of the No_Pretraining model is 3.6361, 195.1% higher than DDLNet (1.2323). However, on Tube 5, the No_Pretraining model’s R^2^ reached 0.7980, close to DDLNet’s 0.8576, indicating that the impact of the pre-training strategy varies across different datasets.

Regarding the contribution of the RSA-BiLSTM branch, the variant model that only retained this branch showed inconsistent performance across different datasets. The RSA variant has an average MSE of 6.5116 and an average MAE of 0.7711, higher than the complete DDLNet. On Tube 2, the RSA variant’s R^2^ value reaches 0.6857, close to DDLNet, but on Tube 1, the R^2^ value is only 0.4081, 40.6% lower than DDLNet’s 0.6866. On Tube 5, the RSA variant’s MAE is 1.5266, 118.4% higher than DDLNet’s 0.6989, indicating that using the RSA-BiLSTM branch alone has limited adaptability to different feature patterns.

The loss function design also has a significant impact on prediction performance. The Simplified_Loss variant has an average MSE of 5.3374 and an average MAE of 0.6119, 90.5% and 53.8% higher than DDLNet, respectively. On Tube 1 and Tube 6, the Simplified_Loss model’s MSE is 8.0198 and 6.8265, 86.3% and 73.8% higher than DDLNet, respectively. Notably, on Tube 5, the Simplified_Loss model’s R^2^ reaches 0.7651, indicating that standard MSE loss can also achieve good results on certain datasets.

Regarding the contribution of the TCN branch, the variant model that only retained the TCN branch has an average MSE of 5.0932 and an average MAE of 0.5983. Its performance is also inconsistent across datasets. For instance, on Tube 5, the TCN variant’s R^2^ is 0.7813, close to DDLNet, while on Tube 1, R^2^ is only 0.4341, significantly lower than DDLNet’s 0.6866. The MSE of the TCN variant on Tube 1 is 7.7832, 80.6% higher than DDLNet, indicating that the capability of the TCN structure varies when processing different temporal features.

Figure 4 shows the comparison of prediction results between the complete DDLNet model and various variant models on the six test datasets. Through visual analysis, it can be seen that the pre-training strategy, RSA-BiLSTM branch, TCN branch, and composite loss function each play different roles in enhancing model performance. The pre-training strategy (as shown in tubes 1 and 4) provides better parameter initialization, significantly reducing prediction fluctuations, with an average MSE reduction of 60.7%. The RSA-BiLSTM branch (Tube 2) excels at capturing global dependencies in temporal data with its residual self-attention mechanism, achieving an R^2^ of 0.6857 on Tube 2. The TCN branch (Tubes 5 and 6) provides multi-scale feature extraction capability through multi-layer dilated convolutions, performing well in handling smooth change regions, with an R^2^ of 0.7813 on Tube 5. The composite loss function (tubes 3 and 5) improves the sensitivity of the model to low RUL value regions by combining absolute and relative errors, reducing MSE by 86.3% and 73.8% on Tube 1 and Tube 6, respectively.

To further validate the rationality of our loss function design, we conducted a comprehensive sensitivity analysis on the dynamic weighting mechanism of the triplet mixed loss function. Using leave-one-out cross-validation across all six CT tube datasets, we systematically evaluated the impact of weight variations on model performance. Our experimental design included the following.

(1) Individual parameter sensitivity testing by varying each weight parameter while keeping others constant;

(2) Parameter interaction analysis through simultaneous variations;

(3) Extreme boundary testing to assess model robustness limits.

As demonstrated in Table 4, our empirically selected configuration (α_base = 0.8, β_base = 0.15, γ_base = 0.05) achieves optimal performance across all evaluation metrics. The individual parameter sensitivity analysis reveals distinct characteristics: α_base exhibits the highest sensitivity, with performance degrading significantly when deviating from 0.8 (MSE increases from 2.92 to 3.45 when α_base = 0.6, representing an 18.2% deterioration). β_base shows moderate sensitivity, with MSE increasing to 3.28 and 3.19 when changed to 0.10 and 0.20, respectively. γ_base demonstrates the least individual impact, though still meaningful, with MSE rising to 3.12 and 3.25 for values of 0.03 and 0.07. The parameter interaction analysis (rows 10–11) shows that simultaneous moderate changes can maintain reasonable performance, while extreme combinations (rows 12–13) result in substantial performance degradation, with MSE increasing by up to 33.2%. This systematic analysis validates that our weight selection represents a well-optimized configuration that effectively balances the contributions of base value loss, phase classification loss, and smoothness regularization.

The collaborative effect of these components enables DDLNet to demonstrate excellent prediction accuracy and stability across datasets with different characteristics, confirming the effectiveness and necessity of the multi-component collaborative design.

### 4.4. Discussion

While our experimental results demonstrate the effectiveness of DDLNet on real CT tube data, several important directions warrant further investigation for practical deployment:

(1) Domain Adaptation and Transferability

Future work should focus on enhancing the model’s adaptability across different CT scanner manufacturers, tube types, and operational environments. The development of domain adaptation techniques could enable our approach to generalize effectively to new hospital settings with minimal retraining, addressing the inherent variability in medical equipment configurations and usage patterns.

(2) Practical System Integration and Deployment

Translating our research findings into operational clinical systems requires comprehensive engineering efforts. This includes developing robust data pipelines for continuous parameter collection, implementing real-time prediction algorithms with appropriate failover mechanisms, establishing integration protocols with existing hospital information systems, and conducting extensive validation studies across multiple institutions to ensure reliability and safety standards.

(3) Model Interpretability and Clinical Trust

A critical aspect for medical equipment applications is enhancing model interpretability to build clinical trust and support regulatory approval. Future research should explore explainable AI techniques that can provide clear reasoning for RUL predictions, develop uncertainty quantification methods to communicate prediction confidence, and establish clinical decision support frameworks that integrate predictions with existing maintenance protocols while maintaining human oversight and accountability.

## 5. Conclusions

This paper addresses the challenge of RUL prediction of CT X-ray tubes by an improved DDLNet model. The architecture employs an adaptive phase-aware framework that combines RSA-BiLSTM and D-TCN and further introduces a dynamic phase detector and triplet mixed loss function to guide the training process. This approach effectively overcomes the limitations of traditional single models in handling the multi-phase variation characteristics of CT tubes.

Through rigorous LOOCV evaluation, experimental results show that DDLNet significantly outperforms models such as LSTM and Transformer across six different CT tube datasets, with an average 2.92 MSE, 0.46 MAE, and 0.77 R^2^ values. This enables healthcare institutions to obtain reliable predictions of CT tube remaining life, helping them formulate scientific maintenance plans.

While our approach demonstrates excellent prediction accuracy and stability, several limitations must be acknowledged, including the purely data-driven nature limiting interpretability and single-institution evaluation constraining generalizability claims. As detailed in Section 4.4, future work should focus on hybrid modeling approaches, multi-institutional validation, and enhanced model interpretability to advance practical clinical deployment. Despite these limitations, the collaborative design of multiple components confirms the effectiveness of the multi-component approach for improving X-ray tube remaining life prediction accuracy.

## Figures and Tables

**Table 1:** X-ray tube information.

CT Number	Tube Number	Start Time	End Time	Data Volume
CT-1	Tube-1	5 August 2021	8 April 2023	223,346
CT-2	Tube-2	26 July 2019	27 June 2023	368,026
CT-3	Tube-3	10 March 2021	23 September 2023	342,590
CT-4	Tube-4	19 July 2019	19 July 2022	401,173
CT-5	Tube-5	1 November 2022	21 July 2023	101,592
CT-6	Tube-6	16 March 2017	2 July 2018	315,587

**Table 2:** Comparative Experimental Results (LOOCV).

		Tube 1	Tube 2	Tube 3	Tube 4	Tube 5	Tube 6	Average	Mean ± Std
LSTM	MSE	7.0079	5.5311	7.2104	6.9019	30.6251	7.9749	10.8752	10.88 ± 9.42
	MAE	1.0671	0.9354	0.8343	1.0600	2.0037	1.5975	1.2497	1.25 ± 0.42
	R^2^	0.4904	0.4202	0.2181	0.1665	0.0018	0.2406	0.2563	0.26 ± 0.18
Transformer	MSE	9.8224	6.7637	8.0440	7.9642	25.6668	8.8649	11.1877	11.19 ± 6.89
	MAE	1.3350	1.0859	1.2381	1.0490	1.8629	1.6177	1.3648	1.36 ± 0.31
	R^2^	0.2858	0.2910	0.1277	0.0383	0.2286	0.1558	0.1879	0.19 ± 0.09
CNN-BiLSTM-Attention	MSE	7.9168	6.9328	7.2402	6.5597	28.7354	8.3603	10.9575	10.96 ± 8.44
	MAE	1.3342	0.8737	1.0731	0.5802	2.1097	1.3533	1.2207	1.22 ± 0.51
	R^2^	0.4243	0.2732	0.2148	0.2079	0.0643	0.2039	0.2314	0.23 ± 0.11
CNN-LSTM	MSE	11.0675	7.1686	8.3275	6.5126	27.3177	7.5794	11.3289	11.33 ± 7.75
	MAE	0.7506	0.7373	0.8901	0.5626	1.8493	1.1650	0.9925	0.99 ± 0.43
	R^2^	0.1952	0.2485	0.0969	0.2132	0.1096	0.2782	0.1903	0.19 ± 0.07
CNN-LSTM-Attention	MSE	10.1311	4.8093	8.1599	6.9712	28.0831	8.9272	11.1803	11.18 ± 8.35
	MAE	0.8454	1.1660	1.4669	0.9929	1.8041	1.0507	1.221	1.22 ± 0.36
	R^2^	0.2633	0.4958	0.1151	0.1582	0.0846	0.1499	0.2112	0.21 ± 0.14
DDLNet	MSE	4.3101	1.4099	2.2568	1.2323	4.3685	3.9320	2.9183	2.92 ± 1.39
	MAE	0.5401	0.2618	0.4395	0.2396	0.6989	0.6072	0.4645	0.46 ± 0.18
	R^2^	0.6866	0.8522	0.7553	0.8512	0.8576	0.6256	0.7714	0.77 ± 0.10

**Table 3:** Ablation experimental results.

		Tube 1	Tube 2	Tube 3	Tube 4	Tube 5	Tube 6	Average
No_ Pretraining	MSE	5.6894	3.0773	3.8784	3.6361	6.1974	4.2178	4.4494
	MAE	0.7138	0.4869	0.5031	0.3441	0.7910	0.6226	0.5769
	R^2^	0.5863	0.6774	0.5794	0.5609	0.7980	0.5983	0.6334
RSA	MSE	8.1404	2.9982	5.0797	1.9206	14.9232	6.2074	6.5449
	MAE	0.8099	0.4659	1.0019	0.3161	1.5266	0.9060	0.8377
	R^2^	0.4081	0.6857	0.4491	0.7681	0.5136	0.4089	0.5389
Simplified_Loss	MSE	8.0198	2.6110	5.6173	2.6930	7.2068	6.8265	5.4957
	MAE	1.0629	0.4379	0.5650	0.4537	0.7515	0.8406	0.6853
	R^2^	0.4168	0.7263	0.3908	0.6748	0.7651	0.3154	0.5482
TCN	MSE	7.7832	2.6779	4.8627	2.3752	6.7110	6.2493	5.1099
	MAE	1.0474	0.5253	0.6724	0.2997	0.7228	0.7227	0.6651
	R^2^	0.4341	0.7193	0.4727	0.7132	0.7813	0.4049	0.5876
Ours	MSE	4.3101	1.4099	2.2568	1.2323	4.3685	3.9320	2.9183
	MAE	0.5401	0.2618	0.4395	0.2396	0.6989	0.6072	0.4645
	R^2^	0.6866	0.8522	0.7553	0.8512	0.8576	0.6256	0.7714

**Table 4:** Sensitivity analysis results on loss function weights (average LOOCV performance).

α_Base	β_Base	γ_Base	Average MSE	Average MAE	Average R^2^
0.8	0.15	0.05	2.92 ± 1.39	0.46 ± 0.18	0.77 ± 0.10
0.6	0.15	0.05	3.45 ± 1.52	0.53 ± 0.19	0.70 ± 0.13
0.7	0.15	0.05	3.08 ± 1.42	0.48 ± 0.17	0.75 ± 0.11
0.9	0.15	0.05	3.15 ± 1.44	0.49 ± 0.17	0.74 ± 0.12
1.0	0.15	0.05	3.38 ± 1.50	0.52 ± 0.19	0.71 ± 0.12
0.8	0.10	0.05	3.28 ± 1.47	0.50 ± 0.18	0.73 ± 0.12
0.8	0.20	0.05	3.19 ± 1.45	0.49 ± 0.17	0.74 ± 0.11
0.8	0.15	0.03	3.12 ± 1.43	0.48 ± 0.17	0.75 ± 0.11
0.8	0.15	0.07	3.25 ± 1.48	0.51 ± 0.19	0.72 ± 0.12
0.7	0.10	0.05	3.42 ± 1.51	0.52 ± 0.19	0.71 ± 0.13
0.9	0.20	0.05	3.56 ± 1.54	0.54 ± 0.20	0.68 ± 0.14
0.6	0.20	0.07	3.89 ± 1.63	0.58 ± 0.22	0.64 ± 0.15
1.0	0.10	0.03	3.71 ± 1.59	0.56 ± 0.21	0.66 ± 0.14

## Data Availability

The original contributions presented in the study are included in the article; further inquiries can be directed to the corresponding author.

## Abbreviations

The following abbreviations are used in this manuscript:

RUL	Remaining Useful Life
CT	Computed Tomography
PdM	Predictive Maintenance
PvM	Preventive Maintenance
IoMT	Internet of Medical Things
DDLNet	Dual-branch Deep Learning Network
RSA-BiLSTM	Residual self-attention Bidirectional Long Short-Term Memory
D-TCN	Dilation-Temporal Convolutional Network
CNN	Convolutional Neural Network
LSTM	Long Short-Term Memory
QKV	Query-Key-Value
MSE	Mean Squared Error
MAE	Mean Absolute Error
R^2^	Coefficient of Determination
EMA	Exponential Moving Average
SVR	Support Vector Regression
CLSTM	Convolutional Long Short-Term Memory
AEC	Automatic Exposure Control
ZEC	Z-direction Exposure Control
LOOCV	Leave-One-Out Cross-Validation
